# Lack of Association between miRNA-146a rs2910164 and miRNA-499 rs3746444 Gene Polymorphisms and Susceptibility to Pulmonary Tuberculosis

**Published:** 2015

**Authors:** Mohammad Naderi, Mohammad Hashemi, Parisa Khorgami, Maliheh Koshki, Mahboubeh Ebrahimi, Shadi Amininia, Batool Sharifi-Mood, Mohsen Taheri

**Affiliations:** 1 *Research Center for Infectious Diseases and Tropical Medicine, Zahedan University of Medical Sciences, Zahedan, Iran.*; 2 *Department of Clinical Biochemistry, School of Medicine, Zahedan University of Medical Sciences, Zahedan, Iran.*; 3 *Department of Internal Medicine, School of Medicine, Zahedan University of Medical Sciences, Zahedan, Iran.*; 4 *Genetics of Non-Communicable Diseases Research Center, Zahedan University of Medical Sciences, Zahedan, Iran.*

**Keywords:** Tuberculosis, microRNA, miRNA-146a, miRNA-499, polymorphism

## Abstract

Single-nucleotide polymorphisms (SNPs) in miRNAsmay alter its expression levels or processing and contribute to susceptibility to a wide range of diseases. Our study aimed to evaluate the possible association between miRNA-146a rs2910164 and miRNA-499 rs3746444 polymorphisms and susceptibility to pulmonary tuberculosis (PTB) in a sample of Iranian population. This case- control study was performed on 202 PTB patients and 204 healthy individuals. Genotyping was performed using tetra amplification refractory mutation system-polymerase chain reaction (T-ARMS-PCR). The results indicated that neither miRNA-499 rs3746444 nor miRNA-146a rs2910164 are associated with the risk of PTB in a sample of Iranian population. Larger studies with different ethnicities are required to validate our findings.

A Tuberculosis, caused by Mycobacterium tuberculosis, remains a major challenge to global public health (1). According to the World Health Organization, the latest estimates included in this report are that there were 8.6 million new TB cases in 2012 and 1.3 million TB deaths.(2). Approximately one third of the world’s population is thought to have been infected with Mycobacte-rium tuberculosis, but only 10% of those develop clinical disease during their lifespan. The precise reasons why only some of the individuals exposed to M. tuberculosis develop disease and others eradicate or limit the disease are unclear. The risk of developing tuberculosis is influenced by multiple factors such as environmental factors, host- pathogen interactions and genetic factors (3, 4).

MicroRNAs (miRNAs) are small non-coding RNAs of 18-23 nucleotides, and their function in cellular physiology, development, and disease is to negatively regulate the expression of protein-coding genes (5). It has been proposed that binding of specific miRNA to the 3′UTR of its target mRNA inhibits gene expression (6).

Previous studies revealed the association between miR-499 rs3746444 as well as miRNA-146a rs2910164 polymorphisms and various diseases susceptibility (6, -, 11, 12). There is only one report regarding the impact of miR-499 rs3746444 and miRNA-146a rs2910164 and susceptibilty to tuberculosis in Chinese Tibetan and Han population (13).The findings show conflicting results so that both the G allele (rs2910164) and the C allele (rs3746444) play different roles in 2 populations (13). In the present study, we aimed to examine the possible association between rs3746444 and rs2910164 polymorphism on risk of PTB in a sample of Iranian population.

## Materials and methods

**Subjects**

This case- control study was performed on 202 PTB patients and 204 population- based healthy subjects. The subjects who underwent PTB treatment and newly diagnosed PTB cases were enrolled in the case group. The diagnosis of PTB was based on clinical, radiological, sputum acid fast bacillus (AFB) smear positivity, culture, and response to antituberculosis therapy as described previously (14, 15). All control subjects were unrelated to patients and from the same geographical origin and living in the same region as the patients with PTB (Zahedan, southeast Iran). Control subjects were unrelated to each other as well as to the patients and selected from the Zahedan population who participated in the metabolic syndrome project and have had no recent signs, symptoms or history of pulmonary infections. The local Ethics Committee of the Zahedan University of Medical Sciences approved the project, and written informed consent was taken from all individuals. Genomic DNA was extracted from whole blood as described previously (16).

**Table 1:** Primers sequence for detection of hsa-miRNA-499 rs3746444 and hsa-miRNA-146a rs2910164 gene polymorphisms.

**Primers**	**Sequence (5`->3`)**	**Amplicon size (bp)**
miR-499 rs3746444 T/C		
FO	GAGTGACCAGGCCCCTTGTCTCTATTAG	422
RO	TTGCTCTTTCACTCTCATTCTGGTGATG	
FI (C allele)	ATGTTTAACTCCTCTCCACGTGACCG	206
RI (T allele)	GGGAAGCAGCACAGACTTGCTGTTAT	268
mir-146a rs2910164 G/C		
FO	GGCCTGGTCTCCTCCAGATGTTTAT	364
RO	ATACCTTCAGAGCCTGAGACTCTGCC	
FI (C allele)	ATGGGTTGTGTCAGTGTCAGACGTC	169
RI (G allele)	GATATCCCAGCTGAAGAACTGAATTTGAC	249

FO: forward outer; RO: reverse outer, FI: forward inner; RI: reverse inner

**Table 2:** Genotypic and allelic frequencies of miRNA-499 rs3746444 T/C in pulmonary tuberculosis (PTB) patients and control groups

miRNA-499 rs3746444 T/C	PTB n (%)	Control n (%)	*OR (95%CI)	P-value
Codominant				
TT	113 (55.9)	117 (57.4)	1.00	-
TC	81 (40.1)	81 (39.7)	1.01 (0.67-1.53)	0.963
CC	8 (4.0)	6 (2.9)	2.01 (0.63-6.54)	0.247
Dominant				
TT	113 (55.9)	117 (57.4)	1.00	-
TC+CC	89 (44.1)	87 (42.6)	1.06 (0.71-1.59)	0.770
Recessive				
TT+TC	194 (96.0)	198 (90.0)	1.001	-
CC	8 (4.0)	6 (2.9)	2.00 (0.66-6.43)	0.244
Alleles				
T	307 (76.0)	315 (77.2)	1.00	-
C	97 (24.0)	93 (22.8)	1.07 (0.77-1.48)	0.740

* Adjusted for Sex and age

**Genotyping**

The genotyping of pre- miRNA-146a rs2910164 and pre-miRNA-499 rs3746444 was done using Tetra amplification refractory mutation system- polymerase chain reaction (T-ARMS-PCR) as described previously (12, 17, 18). The primers are listed in table 1.

**Statistical analyses**

Statistical analysis was done using statistical package SPSS 18 software (SPSS for Windows, SPSS Inc., IL, USA). Data were analyzed by independent sample t-test and χ2 test. The associations between mir-146a as well as mir-499 polymorphisms and PTB were assessed by computing the odds ratio (OR) and 95% confidence intervals (95% CI) from logistic regression analysis adjusted for sex and age. A p-value< 0.05 was considered statistically significant. We estimated the Hardy- Weinberg equilibrium (HWE) separately for cases and controls.

## Results

The study group consisted of 202 PTB patients (72 males and 130 females) with an average age of 50.5± 20.3 years and 204 healthy subjects (94 males and 110 females) with a mean age of 47.3± 15.6 years. No significant difference was found between the groups regarding age (P= 0.072). However, a significant difference was found between the groups concerning sex (P= 0.034). The frequency distribution of miRNA- 499 rs3746444 T/C genotypes in PTB patients and normal subjects is shown in table 2. There was no significant difference between cases and controls concerning rs3746444 T/C polymorphism (χ2= 0.188, P= 0.664). The hsa- miR-499 rs3746444 T/C polymorphism was not a risk/ protection factor for susceptibility to PTB in codominant, dominant and recessive tested inheritance models (Table 2). Furthermore, the rs3746444 C allele was not a risk factor for susceptibility to PTB (OR= 1.07, 95% CI= 0.77- 1.48, P= 0.740).

The genotypes and allele frequencies of miRNA-146a rs2910164 were not significantly different between the PTB patients and the control subjects (χ2= 0.296, P= 0.586). The results showed that miRNA-146a rs2910164 variant was not a risk/ protection factor for predisposition to PTB in codominant, dominant and recessive tested inheritance models (Table 3). In addition, the rs2910164 C allele was not associated with PTB (OR= 0.92, 95% CI= 0.67-1.25, P= 0.631).

**Table 3:** Genotypic and allelic frequencies of miRNA-146a rs2910164 G/C in PTB patients and control groups

**miRNA-146a rs2910164 G/C**	**PTB** **n (%)**	**Control** **n (%)**	* **OR (95% CI)**	**P- value**
Codominant				
GG	116 (57.4)	109 (53.4)	1.00	-
GC	70 (34.7)	80 (39.2)	0.85 (0.56- 1.31)	0.465
CC	16 (7.9)	15 (7.4)	1.02 (0.47- 2.21)	0.951
Dominant				
GG	116 (57.4)	109 (53.4)	1.00	-
GC+CC	86 (42.6)	95 (46.6)	0.88 (0.60- 1.32)	0.537
Recessive				
GG+GC	186 (92.1)	189 (92.6)	1.00	-
CC	16 (7.9)	15 (7.4)	1.09 (0.52- 2.31)	0.819
Alleles				
G	302 (74.7)	298 (73.0)	1.00	-
C	102 (25.3)	110 (27.0)	0.92 (0.67- 1.25)	0.631

* Adjusted for sex and age

## Discussion

In the present study, we examined the possible association of miRNA-146a rs2910164 and miRNA-499 rs3746444 polymorphisms with susceptibility to PTB in a sample of Iranian population. We found no association between the miRNA-146a rs2910164 as well as miRNA-499 rs3746444 polymorphisms and the risk of PTB in our population. Li et al. (13) have found no association between miRNA-499 rs3746444 variant and PTB risk (P= 0.118) in the Han population, but subjects carrying the C allele showed decreased PTB risk (OR=0.403, 95% CI= 0.278- 0.583).While, they found an association between mir- 499 rs3746444 polymorphism and PTB risk (P= 0.022) in Tibetan population and the C allele increased the risk of PTB (OR= 1.870, 95% CI= 1.218- 2.871).

Regarding the mir-146a rs2910164 G>C polymorphism, they found an association between this variant and PTB risk in both Tibetan (P= 0.031) and Han (P< 0.0001) populations. The rs2910164 G allele increased the risk of PTB in Tibetan population (OR= 1.509, 95% CI= 1.100- 2.068), but decreased the PTB risk in Han population (OR= 0.577, 95% CI= 0.452- 0.731). The G allele (rs2910164) plays different roles in 2 populations, as does the C allele (rs3746444) (13). Different lines of evidence show that miRNAs play a key role in host- pathogen interactions. Interferon-**γ** (IFN-**γ**) has a significant function in immune responses to intracellular bacterial infection (19). It has been shown that miR-29 represses immune responses to intracellular pathogens by targeting IFN-**γ** mRNA (20). Toll- like receptors (TLRs) are critical receptors involved in the immune response to many pathogen- related molecules (15). Recently, we have found an association between Toll- interleukin1 receptor (TIR) domain containing adaptor protein (TIRAP) which provides signalling specificity for Toll- like receptors and risk of PTB (21). Besides, it has been suggested that the variants within miRNAs (miRNA-499 rs3746444 and miRNA-499 rs3746444) are regulating the TLRs- mediating signal pathway (13).

It had shown that miRNA profiles in sputum were considerably changed throughout TB infection, which offerd a reason for studying the role of miRNAs in the active pulmonary TB pathogenesis and possibly improve diagnostic, prognostic and therapeutic strategies in the future (22).

MiRNA genetic variants might alter a wide spectrum of biological processes by affecting the processing and/ or selecting their targets (23). An association between miRNA-499 rs3746444 and variety of diseases including breast cancer (7), cervical squamous cell carcinoma (CSCC) (8), hepatocellular carcinoma (9), rheumatoid arthritis(10, 17), coronary artery disease (CAD) (13), Chronic obstructive pulmonary disease (COPD) (24), autoimmune disease (25) and tuberculosis (24) have been reported. Though, no significant association was found between hsa-miRNA-499 rs3746444 and risk of several diseases including SLE (13), schizophrenia (26), asthma (27), colorectal cancer (28), gall bladder (29), breast cancer (30, 18), gastric cancer (31) and lung cancer (32, 33).

It has been shown that miRNA-146a rs2910164 polymorphism was associated with the risk of autoimmune disease (25), coronary artery disease risk (34), severe sepsis (11), acute lymphoblastic leukemia (12). While no association was observed between miRNA-146a rs2910164 and immune thrombocytopenia(35), rheumatoid arthritis (17), digestive tumors (36), colorectal cancer (37), gastric cancer (31) and lung cancer (38).

In conclusion, our findings indicate no significant association between miRNA-499 rs3746444 as well as miRNA-146a rs2910164 gene variants and risk/ protection of pulmonary tuberculosis in a sample of Iranian population.
